# 4-[2,3-Dibromo-3-(4-bromo­phen­yl)propano­yl]-2-phenyl-1,2,3-oxadiazol-2-ium-5-olate

**DOI:** 10.1107/S1600536810040493

**Published:** 2010-10-20

**Authors:** Hoong-Kun Fun, Tara Shahani, Nithinchandra, Balakrishna Kalluraya

**Affiliations:** a X-ray Crystallography Unit, School of Physics, Universiti Sains Malaysia, 11800 USM, Penang, Malaysia; b Department of Studies in Chemistry, Mangalore University, Mangalagangotri, Mangalore 574 199, India

## Abstract

In the title compound, C_17_H_11_Br_3_N_2_O_3_, the whole mol­ecule is disordered over two positions with a refined occupancy ratio of 0.770 (5):0.230 (5). In the major component, the 1,2,3-oxadiazo­lidine ring is essentially planar [maximum deviation = 0.017 (6) Å] and makes dihedral angles of 22.5 (3) and 70.2 (3)° with the 4-bromo­phenyl and phenyl rings, respectively. In the minor component, the corresponding values are 18.9 (11) and 84.9 (12)°. In the crystal, inter­molecular C—H⋯Br hydrogen bonds link the mol­ecules into ribbons along [010]. There is a short O⋯N contact [2.83 (3) Å] in the minor component. In the major component, the mol­ecular structure is stabilized by an intra­molecular C—H⋯O hydrogen bond, which forms an *S*(6) ring motif.

## Related literature

For biological activity of sydnones, mesoionic compounds having a 1,2,3-oxadiazole skeleton and bearing an oxygen atom attached to the 5-position, see: Jyothi *et al.* (2008 ▶); Rai *et al.* (2007 ▶; 2008 ▶). For a related structure, see: Goh *et al.* (2010 ▶). For the stability of the temperature controller used for the data collection, see: Cosier & Glazer (1986 ▶). For bond-length data, see: Allen *et al.* (1987 ▶). For hydrogen-bond motifs, see: Bernstein *et al.* (1995 ▶).
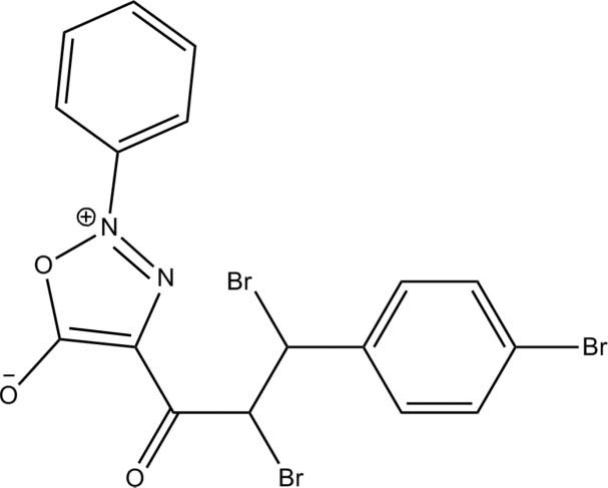


## Experimental

### 

#### Crystal data

C_17_H_11_Br_3_N_2_O_3_
*M* *_r_* = 531.01
Monoclinic, 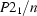

*a* = 17.6996 (3) Å
*b* = 5.8322 (1) Å
*c* = 18.2445 (3) Å
β = 105.973 (1)°
*V* = 1810.62 (5) Å^3^
*Z* = 4
Mo *K*α radiation
μ = 6.70 mm^−1^
*T* = 100 K
0.43 × 0.38 × 0.12 mm

#### Data collection

Bruker SMART APEXII CCD area-detector diffractometer
Absorption correction: multi-scan (*SADABS*; Bruker, 2009 ▶) *T*_min_ = 0.159, *T*_max_ = 0.505
20140 measured reflections
5243 independent reflections
4218 reflections with *I* > 2σ(*I*)
*R*_int_ = 0.028

#### Refinement

*R*[*F* ^2^ > 2σ(*F* ^2^)] = 0.025
*wR*(*F* ^2^) = 0.055
*S* = 1.02
5243 reflections
352 parameters
207 restraints
H-atom parameters constrained
Δρ_max_ = 0.50 e Å^−3^
Δρ_min_ = −0.37 e Å^−3^

### 

Data collection: *APEX2* (Bruker, 2009 ▶); cell refinement: *SAINT* (Bruker, 2009 ▶); data reduction: *SAINT*; program(s) used to solve structure: *SHELXTL* (Sheldrick, 2008 ▶); program(s) used to refine structure: *SHELXTL*; molecular graphics: *SHELXTL*; software used to prepare material for publication: *SHELXTL* and *PLATON* (Spek, 2009 ▶).

## Supplementary Material

Crystal structure: contains datablocks global, I. DOI: 10.1107/S1600536810040493/sj5039sup1.cif

Structure factors: contains datablocks I. DOI: 10.1107/S1600536810040493/sj5039Isup2.hkl

Additional supplementary materials:  crystallographic information; 3D view; checkCIF report

## Figures and Tables

**Table 1:** Hydrogen-bond geometry (Å, °)

*D*—H⋯*A*	*D*—H	H⋯*A*	*D*⋯*A*	*D*—H⋯*A*
C10*A*—H10*A*⋯O2*A*	0.98	2.40	3.168 (4)	135
C14*A*—H14*A*⋯Br3*A*^i^	0.93	2.91	3.809 (5)	163

Symmetry code: (i) 
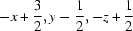
.

**Fig. 1.**
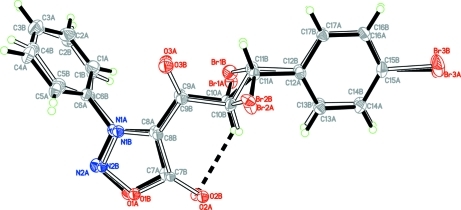
The molecular structure of the title compound, showing 30% probability displacement ellipsoids and the atom-numbering scheme. Both major and minor components are shown. Intramolecular interaction is shown in dashed line.

**Fig. 2.**
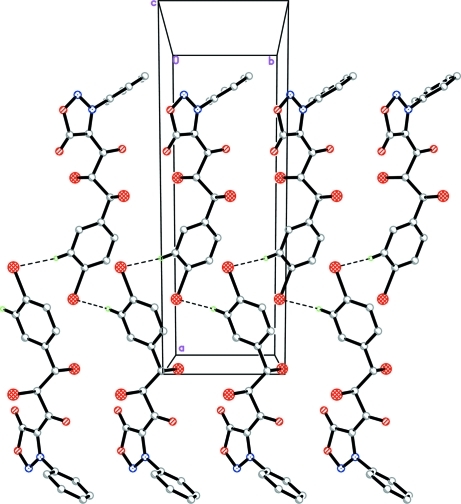
The crystal packing of the title compound, viewed along c axis. Only the major disordered component is shown. Hydrogen atoms not involved in intermolecular hydrogen bonding (dashed lines) are omitted for clarity.

## supplementary crystallographic information

### Comment

Sydnones are mesoionic heterocyclic aromatic chemical compounds. The study of sydnones still remains a field of interests because of their electronic structures and also because of the varied types of biological activities displayed by some of them (Rai *et al.*, 2008). Recently sydnone derivatives were found to exhibit promising antimicrobial properties (Jyothi *et al.*, 2008). Since their discovery, sydnones have shown diverse biological activities and it is thought that the *meso*-ionic nature of the sydnone ring promotes significant interactions with biological systems. Because of wide variety of properties displayed by sydnones we were prompted to synthesize a new chalcone containing a sydnone type ring. Propenones are prepared by the condensation of 4-acetyl-3-arylsydnones with appropriately substituted aromatic aldehydes in an ethanol medium employing sodium hydroxide as catalyst. Bromination of these propenones were carried out using bromine in glacial acetic acid medium to give dibromochalcones (Rai *et al.*, 2007).

In the title compound (Fig. 1), the whole molecule is disordered over two positions with a refined occupancy ratio of 0.770 (5):0.230 (5). This molecule consists of three rings, namely phenyl (C1–C6), 1,2,3-oxadiazolidine (N1/N2/O1/C7/C8) and bromophenyl (C12–C17/Br3) rings. In the major component, the 1,2,3-oxadiazolidine ring is essentially planar (maximum deviation of 0.017 (6) Å at atom N1A) and makes dihedral angles of 22.5 (3) and 70.2 (3)° with 4-bromophenyl and phenyl rings, respectively. In the minor component, the corresponding values are 18.9 (11) and 84.9 (12)° between the 1,2,3-oxadiazolidine ring (maximum deviation of 0.020 (16) Å at atom O1B) and with the 4-bromophenyl and phenyl ring. The bond lengths (Allen *et al.*, 1987) and angles are within normal ranges and are comparable to the closely related structure (Goh *et al.*, 2010). The molecular structure is stabilized by an intramolecular C10A–H10A···O2A hydrogen bond, which forms an S(6) ring motif.

In the crystal packing (Fig. 2 & Fig. 3), intermolecular C14A—H14A···Br3A hydrogen bonds (Table 1), link the molecules into one-dimensional ribbons along the [010] direction. There is a short contact [O2B···N2B = 2.83 (3) Å, symmetry code 1/2 - *x*, -1/2 + *y*, 1/2 - *z*] in the minor component.

### Experimental

1-(3-Phenylsydnon-4yl)-3-(*p*-bromophenyl)-propen-1-one (0.01 mol) was dissolved in glacial acetic acid (25–30 ml) by gentle warming. A solution of bromine in glacial acetic acid (30% *w*/*v*) was added to it with constant stirring till the yellow colour of the bromine persisted. The reaction mixture was stirred at room temperature for 1–2 h. The separated solid was filtered, washed with methanol and dried. It was then recrystallized from ethanol. Crystals suitable for X-ray analysis were obtained from 1:2 mixtures of DMF and ethanol by slow evaporation.

### Refinement

All the H atoms were positioned geometrically [C–H = 0.93 to 0.98 Å] and were refined using a riding model, with *U*_iso_(H) = 1.2 *U*_eq_ (C). The whole molecule is disordered over two positions with a refined ratio of 0.770 (5):0.230 (5). Rigidity, similarity and simulation restraints were applied. The possibility of a supercell in which the whole-molecule disorder would be no longer exit was addressed by examining the *h*0*l*, 0*kl*, *hk*0 precession layers to ensure there are no rows of weak reflections between the rows that represent the current unit cell. No such supercell reflections were found. This finding is consistent with the fact that if such a supercell exists, the occupanies of the major and minor components would be the same. However the refined occupanies are 0.770 (5): 0.230 (5) disproving the existence of a supercell.

### Crystal data

**Table:**

C_17_H_11_Br_3_N_2_O_3_	*F*(000) = 1024
*M**_r_* = 531.01	*D*_x_ = 1.948 Mg m^−^^3^
Monoclinic, *P*2_1_/*n*	Mo *K*α radiation, λ = 0.71073 Å
Hall symbol: -P 2yn	Cell parameters from 9198 reflections
*a* = 17.6996 (3) Å	θ = 2.4–29.8°
*b* = 5.8322 (1) Å	µ = 6.70 mm^−^^1^
*c* = 18.2445 (3) Å	*T* = 100 K
β = 105.973 (1)°	Block, yellow
*V* = 1810.62 (5) Å^3^	0.43 × 0.38 × 0.12 mm
*Z* = 4	

### Data collection

**Table:**

Bruker SMART APEXII CCD area-detector diffractometer	5243 independent reflections
Radiation source: fine-focus sealed tube	4218 reflections with *I* > 2σ(*I*)
graphite	*R*_int_ = 0.028
φ and ω scans	θ_max_ = 30.0°, θ_min_ = 1.9°
Absorption correction: multi-scan (*SADABS*; Bruker, 2009)	*h* = −24→24
*T*_min_ = 0.159, *T*_max_ = 0.505	*k* = −8→7
20140 measured reflections	*l* = −24→25

### Refinement

**Table:**

Refinement on *F*^2^	Primary atom site location: structure-invariant direct methods
Least-squares matrix: full	Secondary atom site location: difference Fourier map
*R*[*F*^2^ > 2σ(*F*^2^)] = 0.025	Hydrogen site location: inferred from neighbouring sites
*wR*(*F*^2^) = 0.055	H-atom parameters constrained
*S* = 1.02	*w* = 1/[σ^2^(*F*_o_^2^) + (0.0238*P*)^2^ + 0.3541*P*] where *P* = (*F*_o_^2^ + 2*F*_c_^2^)/3
5243 reflections	(Δ/σ)_max_ = 0.002
352 parameters	Δρ_max_ = 0.50 e Å^−^^3^
207 restraints	Δρ_min_ = −0.37 e Å^−^^3^

### Special details

**Table:**

Experimental. The crystal was placed in the cold stream of an Oxford Cyrosystems Cobra open-flow nitrogen cryostat (Cosier & Glazer, 1986) operating at 100.0 (1) K.
Geometry. All e.s.d.'s (except the e.s.d. in the dihedral angle between two l.s. planes) are estimated using the full covariance matrix. The cell e.s.d.'s are taken into account individually in the estimation of e.s.d.'s in distances, angles and torsion angles; correlations between e.s.d.'s in cell parameters are only used when they are defined by crystal symmetry. An approximate (isotropic) treatment of cell e.s.d.'s is used for estimating e.s.d.'s involving l.s. planes.
Refinement. Refinement of *F*^2^ against ALL reflections. The weighted *R*-factor *wR* and goodness of fit *S* are based on *F*^2^, conventional *R*-factors *R* are based on *F*, with *F* set to zero for negative *F*^2^. The threshold expression of *F*^2^ > σ(*F*^2^) is used only for calculating *R*-factors(gt) *etc*. and is not relevant to the choice of reflections for refinement. *R*-factors based on *F*^2^ are statistically about twice as large as those based on *F*, and *R*- factors based on ALL data will be even larger.

### Fractional atomic coordinates and isotropic or equivalent isotropic displacement parameters (Å^2^)

**Table:**

	*x*	*y*	*z*	*U*_iso_*/*U*_eq_	Occ. (<1)
Br1A	0.39809 (10)	0.0392 (3)	0.03443 (10)	0.03273 (19)	0.770 (5)
Br2A	0.46821 (7)	0.55027 (18)	0.23460 (8)	0.0428 (3)	0.770 (5)
Br3A	0.80250 (8)	0.0292 (2)	0.16887 (6)	0.03402 (19)	0.770 (5)
O1A	0.1949 (3)	−0.0068 (9)	0.1943 (3)	0.0282 (8)	0.770 (5)
O2A	0.32784 (19)	−0.0399 (6)	0.2397 (2)	0.0293 (6)	0.770 (5)
O3A	0.30678 (12)	0.5098 (4)	0.06838 (17)	0.0339 (6)	0.770 (5)
N1A	0.1841 (3)	0.2416 (10)	0.1078 (3)	0.0233 (9)	0.770 (5)
N2A	0.1416 (3)	0.1153 (13)	0.1392 (4)	0.0299 (11)	0.770 (5)
C1A	0.1437 (4)	0.3491 (9)	−0.0250 (3)	0.0435 (13)	0.770 (5)
H1AA	0.1703	0.2222	−0.0363	0.052*	0.770 (5)
C2A	0.1033 (3)	0.4963 (9)	−0.0814 (3)	0.0473 (11)	0.770 (5)
H2AA	0.1010	0.4676	−0.1321	0.057*	0.770 (5)
C3A	0.0659 (3)	0.6880 (8)	−0.0620 (3)	0.0416 (10)	0.770 (5)
H3AA	0.0396	0.7889	−0.1000	0.050*	0.770 (5)
C4A	0.0676 (4)	0.7296 (9)	0.0128 (3)	0.0381 (10)	0.770 (5)
H4AA	0.0414	0.8564	0.0248	0.046*	0.770 (5)
C5A	0.1078 (5)	0.5851 (12)	0.0702 (4)	0.0301 (11)	0.770 (5)
H5AA	0.1109	0.6131	0.1211	0.036*	0.770 (5)
C6A	0.1432 (6)	0.3969 (14)	0.0479 (3)	0.0292 (16)	0.770 (5)
C7A	0.2730 (2)	0.0478 (7)	0.1932 (3)	0.0261 (8)	0.770 (5)
C8A	0.26327 (18)	0.2104 (6)	0.1337 (2)	0.0231 (7)	0.770 (5)
C12A	0.54873 (13)	0.3090 (5)	0.14317 (17)	0.0243 (6)	0.770 (5)
C13A	0.57473 (14)	0.1083 (5)	0.18318 (18)	0.0274 (6)	0.770 (5)
H13A	0.5415	0.0287	0.2057	0.033*	0.770 (5)
C14A	0.6501 (2)	0.0248 (8)	0.1899 (3)	0.0292 (8)	0.770 (5)
H14A	0.6677	−0.1086	0.2172	0.035*	0.770 (5)
C15A	0.6983 (4)	0.1445 (18)	0.1551 (9)	0.033 (2)	0.770 (5)
C16A	0.6744 (3)	0.3462 (14)	0.1168 (5)	0.0332 (15)	0.770 (5)
H16A	0.7085	0.4278	0.0958	0.040*	0.770 (5)
C17A	0.59879 (19)	0.4272 (7)	0.1096 (2)	0.0267 (7)	0.770 (5)
H17A	0.5817	0.5609	0.0823	0.032*	0.770 (5)
C9A	0.32136 (14)	0.3360 (5)	0.10637 (19)	0.0266 (6)	0.770 (5)
C10A	0.40246 (13)	0.2259 (5)	0.12510 (16)	0.0245 (6)	0.770 (5)
H10A	0.4109	0.1284	0.1704	0.029*	0.770 (5)
C11A	0.46785 (13)	0.4009 (4)	0.13558 (15)	0.0241 (6)	0.770 (5)
H11A	0.4542	0.5140	0.0943	0.029*	0.770 (5)
Br1B	0.3986 (4)	0.0778 (11)	0.0224 (4)	0.0432 (10)	0.230 (5)
Br2B	0.4661 (2)	0.5668 (4)	0.2298 (2)	0.0207 (6)	0.230 (5)
Br3B	0.8041 (3)	0.0491 (10)	0.1649 (3)	0.0645 (14)	0.230 (5)
O1B	0.2081 (8)	−0.011 (3)	0.1999 (11)	0.025 (3)*	0.230 (5)
O2B	0.3419 (6)	−0.015 (2)	0.2543 (7)	0.025 (3)*	0.230 (5)
O3B	0.3133 (5)	0.5587 (14)	0.0938 (5)	0.035 (2)*	0.230 (5)
N1B	0.1956 (8)	0.248 (4)	0.1157 (11)	0.025 (4)*	0.230 (5)
N2B	0.1529 (8)	0.109 (4)	0.1467 (12)	0.016 (3)*	0.230 (5)
C1B	0.1377 (11)	0.305 (3)	−0.0245 (9)	0.021 (3)*	0.230 (5)
H1BA	0.1580	0.1639	−0.0332	0.026*	0.230 (5)
C2B	0.0980 (11)	0.437 (2)	−0.0865 (9)	0.035 (4)*	0.230 (5)
H2BA	0.0960	0.3898	−0.1357	0.042*	0.230 (5)
C3B	0.0620 (10)	0.635 (2)	−0.0751 (9)	0.031 (3)*	0.230 (5)
H3BA	0.0310	0.7161	−0.1164	0.037*	0.230 (5)
C4B	0.0718 (13)	0.714 (3)	−0.0023 (10)	0.031 (4)*	0.230 (5)
H4BA	0.0516	0.8565	0.0055	0.037*	0.230 (5)
C5B	0.112 (2)	0.581 (5)	0.0605 (12)	0.038 (6)*	0.230 (5)
H5BA	0.1144	0.6306	0.1095	0.045*	0.230 (5)
C6B	0.1480 (19)	0.378 (5)	0.0502 (9)	0.022 (5)*	0.230 (5)
C7B	0.2854 (7)	0.064 (3)	0.2074 (7)	0.015 (3)*	0.230 (5)
C8B	0.2729 (6)	0.237 (2)	0.1508 (6)	0.019 (3)*	0.230 (5)
C12B	0.5443 (5)	0.2546 (16)	0.1212 (5)	0.023 (2)*	0.230 (5)
C13B	0.5719 (6)	0.0563 (17)	0.1606 (6)	0.029 (3)*	0.230 (5)
H13B	0.5374	−0.0375	0.1771	0.035*	0.230 (5)
C14B	0.6499 (8)	−0.005 (3)	0.1759 (10)	0.032 (4)*	0.230 (5)
H14B	0.6676	−0.1432	0.2000	0.038*	0.230 (5)
C15B	0.7008 (10)	0.142 (5)	0.155 (3)	0.019 (6)*	0.230 (5)
C16B	0.6757 (10)	0.331 (4)	0.1083 (16)	0.018 (3)*	0.230 (5)
H16B	0.7096	0.4148	0.0875	0.022*	0.230 (5)
C17B	0.5971 (7)	0.388 (2)	0.0948 (8)	0.026 (3)*	0.230 (5)
H17B	0.5786	0.5199	0.0670	0.031*	0.230 (5)
C9B	0.3306 (5)	0.3857 (16)	0.1293 (6)	0.025 (2)*	0.230 (5)
C10B	0.4170 (4)	0.3055 (17)	0.1585 (5)	0.029 (2)	0.230 (5)
H10B	0.4223	0.1566	0.1843	0.035*	0.230 (5)
C11B	0.4576 (4)	0.3139 (16)	0.0966 (5)	0.032 (2)	0.230 (5)
H11B	0.4503	0.4655	0.0725	0.038*	0.230 (5)

### Atomic displacement parameters (Å^2^)

**Table:**

	*U*^11^	*U*^22^	*U*^33^	*U*^12^	*U*^13^	*U*^23^
Br1A	0.0304 (2)	0.0336 (3)	0.0315 (5)	−0.0036 (2)	0.0040 (3)	−0.0067 (3)
Br2A	0.0259 (3)	0.0618 (6)	0.0411 (4)	−0.0048 (3)	0.0101 (3)	−0.0172 (4)
Br3A	0.0247 (4)	0.0442 (3)	0.0324 (3)	0.0101 (2)	0.0065 (2)	−0.0044 (2)
O1A	0.0256 (15)	0.0239 (12)	0.0370 (18)	0.0000 (14)	0.0119 (15)	0.0039 (9)
O2A	0.0259 (13)	0.0293 (13)	0.0317 (16)	0.0041 (11)	0.0061 (11)	0.0065 (12)
O3A	0.0259 (10)	0.0315 (12)	0.0420 (15)	0.0016 (8)	0.0054 (10)	0.0170 (11)
N1A	0.0199 (14)	0.0208 (13)	0.0299 (18)	−0.0014 (13)	0.0080 (12)	−0.0024 (12)
N2A	0.0267 (19)	0.0269 (15)	0.034 (2)	−0.0022 (17)	0.0050 (17)	−0.0016 (12)
C1A	0.047 (2)	0.040 (3)	0.048 (2)	0.004 (2)	0.0208 (15)	0.0047 (19)
C2A	0.054 (2)	0.056 (3)	0.0329 (18)	0.000 (2)	0.0135 (15)	0.0065 (19)
C3A	0.0350 (17)	0.035 (2)	0.046 (3)	−0.0005 (16)	−0.0032 (15)	0.0080 (18)
C4A	0.0311 (17)	0.0349 (19)	0.043 (3)	0.0083 (11)	0.001 (2)	−0.0001 (18)
C5A	0.0250 (17)	0.0277 (19)	0.034 (2)	0.0012 (10)	0.0011 (19)	−0.0035 (15)
C6A	0.022 (2)	0.028 (3)	0.036 (2)	0.0023 (12)	0.0047 (11)	0.0061 (12)
C7A	0.0251 (17)	0.0225 (14)	0.030 (2)	−0.0001 (12)	0.0070 (14)	−0.0037 (15)
C8A	0.0212 (14)	0.0225 (14)	0.0239 (16)	0.0007 (9)	0.0035 (12)	−0.0004 (13)
C12A	0.0218 (11)	0.0239 (13)	0.0265 (14)	−0.0024 (9)	0.0057 (10)	−0.0035 (11)
C13A	0.0254 (12)	0.0295 (14)	0.0277 (15)	−0.0032 (10)	0.0079 (10)	0.0007 (12)
C14A	0.0269 (15)	0.0287 (17)	0.030 (2)	0.0057 (11)	0.0041 (12)	0.0021 (16)
C15A	0.021 (2)	0.045 (3)	0.030 (2)	0.0018 (11)	0.0034 (11)	−0.0086 (12)
C16A	0.0256 (16)	0.045 (3)	0.032 (3)	−0.0077 (12)	0.0123 (13)	−0.005 (2)
C17A	0.0276 (14)	0.0241 (16)	0.0286 (18)	−0.0012 (10)	0.0083 (12)	0.0004 (15)
C9A	0.0225 (12)	0.0277 (14)	0.0279 (15)	−0.0012 (10)	0.0042 (10)	0.0012 (12)
C10A	0.0218 (11)	0.0253 (14)	0.0247 (14)	−0.0001 (9)	0.0035 (10)	0.0024 (10)
C11A	0.0217 (11)	0.0252 (13)	0.0250 (14)	−0.0001 (9)	0.0054 (9)	0.0019 (10)
Br1B	0.0388 (8)	0.057 (2)	0.0294 (14)	−0.0112 (12)	0.0026 (8)	−0.0042 (12)
Br2B	0.0323 (12)	0.0050 (8)	0.0308 (10)	−0.0039 (6)	0.0186 (8)	−0.0053 (6)
Br3B	0.0315 (16)	0.096 (3)	0.070 (2)	0.0027 (15)	0.0214 (14)	−0.0156 (16)
C10B	0.022 (4)	0.029 (5)	0.033 (5)	−0.002 (3)	0.001 (4)	0.004 (4)
C11B	0.027 (4)	0.032 (5)	0.032 (5)	−0.005 (3)	0.001 (3)	0.001 (4)

### Geometric parameters (Å, °)

**Table:**

Br1A—C10A	1.964 (4)	Br1B—C11B	2.010 (12)
Br2A—C11A	2.004 (3)	Br2B—C10B	2.036 (11)
Br3A—C15A	1.913 (5)	Br3B—C15B	1.867 (13)
O1A—N2A	1.374 (5)	O1B—N2B	1.367 (13)
O1A—C7A	1.423 (5)	O1B—C7B	1.407 (13)
O2A—C7A	1.212 (4)	O2B—C7B	1.214 (12)
O3A—C9A	1.215 (3)	O3B—C9B	1.193 (11)
N1A—N2A	1.293 (4)	N1B—N2B	1.334 (14)
N1A—C8A	1.363 (4)	N1B—C8B	1.344 (12)
N1A—C6A	1.450 (5)	N1B—C6B	1.469 (13)
C1A—C6A	1.362 (5)	C1B—C2B	1.386 (14)
C1A—C2A	1.379 (5)	C1B—C6B	1.392 (13)
C1A—H1AA	0.9300	C1B—H1BA	0.9300
C2A—C3A	1.393 (5)	C2B—C3B	1.365 (14)
C2A—H2AA	0.9300	C2B—H2BA	0.9300
C3A—C4A	1.380 (6)	C3B—C4B	1.370 (14)
C3A—H3AA	0.9300	C3B—H3BA	0.9300
C4A—C5A	1.379 (5)	C4B—C5B	1.403 (15)
C4A—H4AA	0.9300	C4B—H4BA	0.9300
C5A—C6A	1.379 (6)	C5B—C6B	1.382 (14)
C5A—H5AA	0.9300	C5B—H5BA	0.9300
C7A—C8A	1.415 (4)	C7B—C8B	1.419 (12)
C8A—C9A	1.458 (3)	C8B—C9B	1.472 (11)
C12A—C13A	1.390 (3)	C12B—C13B	1.378 (11)
C12A—C17A	1.391 (3)	C12B—C17B	1.400 (12)
C12A—C11A	1.499 (3)	C12B—C11B	1.515 (10)
C13A—C14A	1.393 (4)	C13B—C14B	1.378 (13)
C13A—H13A	0.9300	C13B—H13B	0.9300
C14A—C15A	1.385 (6)	C14B—C15B	1.374 (14)
C14A—H14A	0.9300	C14B—H14B	0.9300
C15A—C16A	1.374 (6)	C15B—C16B	1.389 (14)
C16A—C17A	1.391 (5)	C16B—C17B	1.384 (14)
C16A—H16A	0.9300	C16B—H16B	0.9300
C17A—H17A	0.9300	C17B—H17B	0.9300
C9A—C10A	1.523 (3)	C9B—C10B	1.547 (10)
C10A—C11A	1.515 (3)	C10B—C11B	1.497 (11)
C10A—H10A	0.9800	C10B—H10B	0.9800
C11A—H11A	0.9800	C11B—H11B	0.9800
			
N2A—O1A—C7A	110.4 (4)	N2B—O1B—C7B	113.3 (12)
N2A—N1A—C8A	116.0 (4)	N2B—N1B—C8B	113.0 (11)
N2A—N1A—C6A	117.4 (5)	N2B—N1B—C6B	113.1 (17)
C8A—N1A—C6A	126.6 (5)	C8B—N1B—C6B	133.9 (18)
N1A—N2A—O1A	104.6 (4)	N1B—N2B—O1B	103.6 (12)
C6A—C1A—C2A	117.4 (5)	C2B—C1B—C6B	122.0 (14)
C6A—C1A—H1AA	121.3	C2B—C1B—H1BA	119.0
C2A—C1A—H1AA	121.3	C6B—C1B—H1BA	119.0
C1A—C2A—C3A	119.7 (4)	C3B—C2B—C1B	119.9 (14)
C1A—C2A—H2AA	120.1	C3B—C2B—H2BA	120.1
C3A—C2A—H2AA	120.1	C1B—C2B—H2BA	120.1
C4A—C3A—C2A	120.6 (4)	C2B—C3B—C4B	119.5 (14)
C4A—C3A—H3AA	119.7	C2B—C3B—H3BA	120.2
C2A—C3A—H3AA	119.7	C4B—C3B—H3BA	120.2
C5A—C4A—C3A	120.6 (4)	C3B—C4B—C5B	120.4 (15)
C5A—C4A—H4AA	119.7	C3B—C4B—H4BA	119.8
C3A—C4A—H4AA	119.7	C5B—C4B—H4BA	119.8
C4A—C5A—C6A	116.4 (5)	C6B—C5B—C4B	120.8 (16)
C4A—C5A—H5AA	121.8	C6B—C5B—H5BA	119.6
C6A—C5A—H5AA	121.8	C4B—C5B—H5BA	119.6
C1A—C6A—C5A	125.1 (5)	C5B—C6B—C1B	117.0 (13)
C1A—C6A—N1A	118.2 (5)	C5B—C6B—N1B	121.1 (16)
C5A—C6A—N1A	116.6 (5)	C1B—C6B—N1B	122.0 (16)
O2A—C7A—C8A	136.4 (4)	O2B—C7B—O1B	122.8 (12)
O2A—C7A—O1A	119.3 (4)	O2B—C7B—C8B	135.8 (12)
C8A—C7A—O1A	104.3 (3)	O1B—C7B—C8B	101.4 (10)
N1A—C8A—C7A	104.6 (3)	N1B—C8B—C7B	108.4 (10)
N1A—C8A—C9A	124.7 (3)	N1B—C8B—C9B	122.3 (10)
C7A—C8A—C9A	130.6 (3)	C7B—C8B—C9B	129.3 (10)
C13A—C12A—C17A	119.4 (2)	C13B—C12B—C17B	118.1 (9)
C13A—C12A—C11A	121.3 (2)	C13B—C12B—C11B	122.1 (8)
C17A—C12A—C11A	119.4 (2)	C17B—C12B—C11B	119.3 (9)
C12A—C13A—C14A	120.7 (3)	C14B—C13B—C12B	121.0 (11)
C12A—C13A—H13A	119.6	C14B—C13B—H13B	119.5
C14A—C13A—H13A	119.6	C12B—C13B—H13B	119.5
C15A—C14A—C13A	118.7 (4)	C15B—C14B—C13B	118.6 (13)
C15A—C14A—H14A	120.7	C15B—C14B—H14B	120.7
C13A—C14A—H14A	120.7	C13B—C14B—H14B	120.7
C16A—C15A—C14A	121.5 (5)	C14B—C15B—C16B	122.9 (15)
C16A—C15A—Br3A	121.3 (4)	C14B—C15B—Br3B	119.4 (12)
C14A—C15A—Br3A	117.1 (4)	C16B—C15B—Br3B	115.5 (12)
C15A—C16A—C17A	119.5 (5)	C17B—C16B—C15B	115.8 (14)
C15A—C16A—H16A	120.2	C17B—C16B—H16B	122.1
C17A—C16A—H16A	120.2	C15B—C16B—H16B	122.1
C12A—C17A—C16A	120.2 (4)	C16B—C17B—C12B	122.7 (13)
C12A—C17A—H17A	119.9	C16B—C17B—H17B	118.7
C16A—C17A—H17A	119.9	C12B—C17B—H17B	118.7
O3A—C9A—C8A	123.7 (2)	O3B—C9B—C8B	123.4 (9)
O3A—C9A—C10A	121.3 (2)	O3B—C9B—C10B	121.6 (9)
C8A—C9A—C10A	114.9 (2)	C8B—C9B—C10B	115.0 (8)
C11A—C10A—C9A	112.6 (2)	C11B—C10B—C9B	111.4 (7)
C11A—C10A—Br1A	109.35 (19)	C11B—C10B—Br2B	104.3 (7)
C9A—C10A—Br1A	103.21 (18)	C9B—C10B—Br2B	101.7 (6)
C11A—C10A—H10A	110.5	C11B—C10B—H10B	112.9
C9A—C10A—H10A	110.5	C9B—C10B—H10B	112.9
Br1A—C10A—H10A	110.5	Br2B—C10B—H10B	112.9
C12A—C11A—C10A	116.6 (2)	C10B—C11B—C12B	115.3 (7)
C12A—C11A—Br2A	107.83 (17)	C10B—C11B—Br1B	102.5 (7)
C10A—C11A—Br2A	102.68 (17)	C12B—C11B—Br1B	109.8 (6)
C12A—C11A—H11A	109.8	C10B—C11B—H11B	109.7
C10A—C11A—H11A	109.8	C12B—C11B—H11B	109.7
Br2A—C11A—H11A	109.8	Br1B—C11B—H11B	109.7
			
C8A—N1A—N2A—O1A	3.2 (9)	C8B—N1B—N2B—O1B	−6(3)
C6A—N1A—N2A—O1A	−178.2 (6)	C6B—N1B—N2B—O1B	174 (2)
C7A—O1A—N2A—N1A	−1.8 (9)	C7B—O1B—N2B—N1B	5(3)
C6A—C1A—C2A—C3A	1.7 (10)	C6B—C1B—C2B—C3B	−6(3)
C1A—C2A—C3A—C4A	−1.3 (8)	C1B—C2B—C3B—C4B	6(3)
C2A—C3A—C4A—C5A	1.5 (10)	C2B—C3B—C4B—C5B	−6(4)
C3A—C4A—C5A—C6A	−2.0 (13)	C3B—C4B—C5B—C6B	5(5)
C2A—C1A—C6A—C5A	−2.4 (15)	C4B—C5B—C6B—C1B	−4(6)
C2A—C1A—C6A—N1A	178.8 (7)	C4B—C5B—C6B—N1B	175 (3)
C4A—C5A—C6A—C1A	2.5 (17)	C2B—C1B—C6B—C5B	4(5)
C4A—C5A—C6A—N1A	−178.6 (8)	C2B—C1B—C6B—N1B	−175 (2)
N2A—N1A—C6A—C1A	−109.4 (10)	N2B—N1B—C6B—C5B	84 (4)
C8A—N1A—C6A—C1A	69.1 (12)	C8B—N1B—C6B—C5B	−96 (4)
N2A—N1A—C6A—C5A	71.7 (12)	N2B—N1B—C6B—C1B	−96 (4)
C8A—N1A—C6A—C5A	−109.9 (10)	C8B—N1B—C6B—C1B	83 (4)
N2A—O1A—C7A—O2A	178.0 (6)	N2B—O1B—C7B—O2B	176 (2)
N2A—O1A—C7A—C8A	−0.1 (7)	N2B—O1B—C7B—C8B	−2(2)
N2A—N1A—C8A—C7A	−3.3 (8)	N2B—N1B—C8B—C7B	5(3)
C6A—N1A—C8A—C7A	178.3 (6)	C6B—N1B—C8B—C7B	−175 (2)
N2A—N1A—C8A—C9A	−179.6 (6)	N2B—N1B—C8B—C9B	−176.1 (18)
C6A—N1A—C8A—C9A	2.0 (9)	C6B—N1B—C8B—C9B	4(4)
O2A—C7A—C8A—N1A	−175.7 (6)	O2B—C7B—C8B—N1B	−180 (2)
O1A—C7A—C8A—N1A	1.8 (5)	O1B—C7B—C8B—N1B	−1(2)
O2A—C7A—C8A—C9A	0.3 (9)	O2B—C7B—C8B—C9B	1(3)
O1A—C7A—C8A—C9A	177.9 (4)	O1B—C7B—C8B—C9B	179.6 (15)
C17A—C12A—C13A—C14A	0.0 (5)	C17B—C12B—C13B—C14B	2.2 (16)
C11A—C12A—C13A—C14A	−179.9 (3)	C11B—C12B—C13B—C14B	173.7 (11)
C12A—C13A—C14A—C15A	−0.8 (10)	C12B—C13B—C14B—C15B	4(3)
C13A—C14A—C15A—C16A	2.3 (18)	C13B—C14B—C15B—C16B	−11 (5)
C13A—C14A—C15A—Br3A	177.9 (6)	C13B—C14B—C15B—Br3B	−173 (2)
C14A—C15A—C16A—C17A	−3(2)	C14B—C15B—C16B—C17B	11 (6)
Br3A—C15A—C16A—C17A	−178.4 (8)	Br3B—C15B—C16B—C17B	174 (2)
C13A—C12A—C17A—C16A	−0.7 (7)	C15B—C16B—C17B—C12B	−5(4)
C11A—C12A—C17A—C16A	179.2 (5)	C13B—C12B—C17B—C16B	−2(2)
C15A—C16A—C17A—C12A	2.2 (13)	C11B—C12B—C17B—C16B	−173.4 (18)
N1A—C8A—C9A—O3A	14.4 (6)	N1B—C8B—C9B—O3B	19 (2)
C7A—C8A—C9A—O3A	−160.9 (4)	C7B—C8B—C9B—O3B	−162.4 (13)
N1A—C8A—C9A—C10A	−162.3 (4)	N1B—C8B—C9B—C10B	−163.2 (16)
C7A—C8A—C9A—C10A	22.4 (6)	C7B—C8B—C9B—C10B	15.8 (17)
O3A—C9A—C10A—C11A	34.0 (4)	O3B—C9B—C10B—C11B	−48.6 (13)
C8A—C9A—C10A—C11A	−149.2 (3)	C8B—C9B—C10B—C11B	133.2 (10)
O3A—C9A—C10A—Br1A	−83.8 (3)	O3B—C9B—C10B—Br2B	62.0 (10)
C8A—C9A—C10A—Br1A	93.0 (3)	C8B—C9B—C10B—Br2B	−116.1 (8)
C13A—C12A—C11A—C10A	−37.9 (4)	C9B—C10B—C11B—C12B	175.8 (7)
C17A—C12A—C11A—C10A	142.2 (3)	Br2B—C10B—C11B—C12B	66.9 (8)
C13A—C12A—C11A—Br2A	76.9 (3)	C9B—C10B—C11B—Br1B	−64.9 (8)
C17A—C12A—C11A—Br2A	−103.0 (3)	Br2B—C10B—C11B—Br1B	−173.8 (4)
C9A—C10A—C11A—C12A	−171.6 (2)	C13B—C12B—C11B—C10B	51.9 (13)
Br1A—C10A—C11A—C12A	−57.5 (3)	C17B—C12B—C11B—C10B	−136.7 (10)
C9A—C10A—C11A—Br2A	70.8 (2)	C13B—C12B—C11B—Br1B	−63.2 (10)
Br1A—C10A—C11A—Br2A	−175.09 (13)	C17B—C12B—C11B—Br1B	108.2 (9)

### Hydrogen-bond geometry (Å, °)

**Table:**

*D*—H···*A*	*D*—H	H···*A*	*D*···*A*	*D*—H···*A*
C10A—H10A···O2A	0.98	2.40	3.168 (4)	135
C14A—H14A···Br3A^i^	0.93	2.91	3.809 (5)	163

Symmetry codes: (i) −*x*+3/2, *y*−1/2, −*z*+1/2.
